# Alternative mRNA splicing in anthracycline-induced cardiomyopathy – a COG-ALTE03N1 report

**DOI:** 10.1186/s40959-025-00345-2

**Published:** 2025-05-17

**Authors:** Purnima Singh, David K. Crossman, Changde Cheng, Patrick J. Trainor, Noha Sharafeldin, Xuexia Wang, Liting Zhou, Lindsey Hageman, Saro H. Armenian, Frank M. Balis, Douglas S. Hawkins, Frank G. Keller, Melissa M. Hudson, Joseph P. Neglia, Jill P. Ginsberg, Wendy Landier, Smita Bhatia

**Affiliations:** 1 Institute for Cancer Outcomes and Survivorship, University of Alabama at Birmingham, Birmingham, AL USA; 2 Department of Pediatrics, University of Alabama at Birmingham, Birmingham, AL 35233 USA; 3 Department of Genetics, University of Alabama at Birmingham, Birmingham, AL USA; 4 Department of Biostatistics, Florida International University, Miami, FL USA; 5 Department of Population Sciences, City of Hope, Duarte, CA USA; 6 Department of Pediatrics, Children’s Hospital of Philadelphia, Philadelphia, PA USA; 7 Department of Pediatrics, Seattle Children’s, Seattle, WA USA; 8 Department of Pediatrics, Children’s Healthcare of Atlanta, Emory University, Atlanta, GA USA; 9 Department of Epidemiology and Cancer Control, St. Jude Children’s Research Hospital, Memphis, TN USA; 10 Department of Pediatrics, University of Minnesota, Minneapolis, MN USA

**Keywords:** Alternative splicing, Peripheral blood, Transcriptome, Anthracyclines, Childhood cancer survivors

## Abstract

**Background:**

Anthracycline-induced cardiomyopathy is a well-established adverse consequence in childhood cancer survivors. Altered mRNA expression in the peripheral blood has been found at the level of genes and pathways among anthracycline-exposed childhood cancer survivors with and without cardiomyopathy. However, the role of aberrant alternative splicing in anthracycline-induced cardiomyopathy remains unexplored. The present study examined if transcript-specific events, due to alternative splicing occur in anthracycline-exposed childhood cancer survivors with and without cardiomyopathy.

**Methods:**

Participants were anthracycline-exposed childhood cancer survivors with cardiomyopathy (cases) matched with anthracycline-exposed childhood cancer survivors without cardiomyopathy (controls; matched on primary cancer diagnosis, year of diagnosis, and race/ethnicity). mRNA sequencing was performed on total RNA from peripheral blood in 32 cases and 32 matched controls. Event-level splicing tool, rMATS (replicate Multivariate Analysis of Transcript Splicing) was used for quantitative profiling of alternative splicing events.

**Results:**

A total of 45 alternative splicing events in 36 genes were identified. Using a prioritization strategy to filter the alternative splicing events, intron retention in *RPS24* and skipped exon of *PFND5* showed differential expression of altered transcripts.

**Conclusions:**

We identified specific alternative splicing events in anthracycline-exposed childhood cancer survivors with and without cardiomyopathy. Our findings suggest that differential alternative splicing events can provide additional insight into the peripheral blood transcriptomic landscape of anthracycline-induced cardiomyopathy.

**Graphical abstract:**

Central Illustration. Aberrant alternative splicing and anthracycline-induced cardiomyopathy. This study sought to identify alternative splice variants that are differentially abundant between anthracycline-exposed childhood cancer survivors that developed cardiomyopathy (cases) versus those who did not (controls). We observed dysregulated alternative splicing of *PFDN5* and *RPS24* is associated with the development of cardiomyopathy.Splicing defects in *PFDN5* impair cytoskeletal protein folding, while *RPS24* dysregulation affects their translation, disrupting actin and tubulin homeostasis. Together, these alterations destabilize cardiomyocyte structure, contributing to sarcomere disorganization and the development of cardiomyopathy. Created in BioRender. Singh, P. (2025) https://BioRender.com/59zmgls.

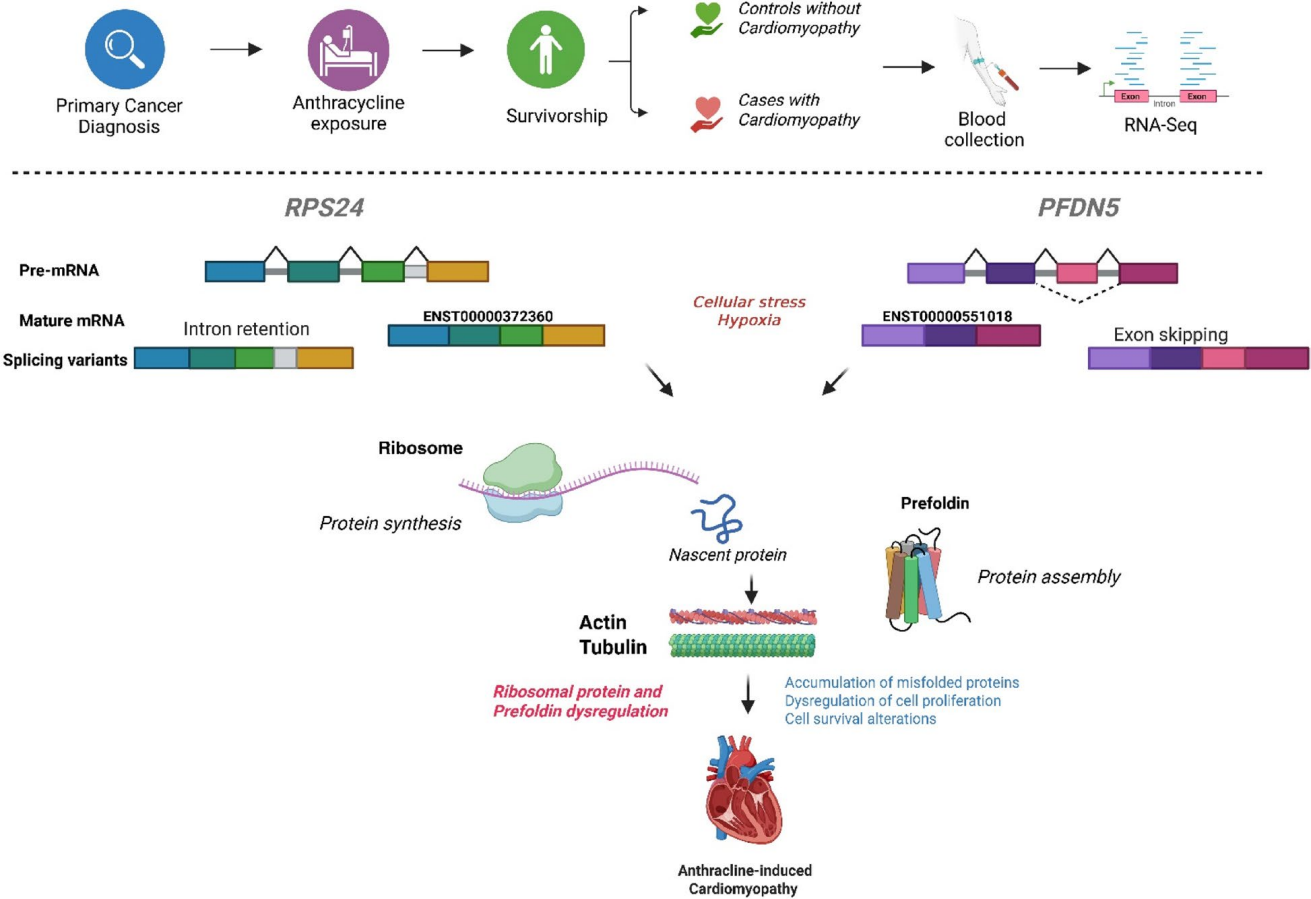

**Supplementary Information:**

The online version contains supplementary material available at 10.1186/s40959-025-00345-2.

## Background

Anthracycline-induced cardiotoxicity represents a continuum from subclinical myocyte injury to congestive heart failure [1]. Studies of differential gene expression (DGE) in anthracycline-exposed cancer survivors employing microarrays [2] and RNA sequencing (RNA-Seq) [3, 4] from peripheral blood collected following cardiomyopathy diagnosis have identified molecular signatures and pathways associated with anthracycline-induced cardiomyopathy. However, DGE studies do not account for mRNA isoform/transcript diversity generated by differential or alternative splicing [5].

mRNA splicing is one of the regulatory mechanisms for gene expression and is essential to the flow of information from the DNA to protein in all eukaryotes [6]. More than 90% of human protein-coding genes produce multiple transcripts through alternative splicing [7, 8], resulting in diversification and expansion of the transcriptome and proteome [9]. Alternatively spliced isoforms may have related, distinct, or even opposing functions or subcellular localizations, or no protein product. There are five basic types of alternative splicing: alternative 5′ splice site (A5'SS), skipped exon (SE), mutually exclusive exons (MXE), retained intron (RI), and alternative 3′ splice site (A3'SS) [8]. Alternative splicing plays a role in biological processes such as cell differentiation and proliferation, organ development and stress response [10]. Aberrant splicing underlies many pathological processes including premature aging, infection, and inflammation, as well as in immune and metabolic disorders and cardiovascular disease [11–13]. Aberrant splicing of sarcomeric and ion channel genes has been seen in patients with cardiomyopathy in non-oncology settings [14–16]. The recent identification of several cardiac splice factors, such as RNA-binding motif protein 20 and 24, has provided insight as to how these splicing factors cause cardiomyopathy [17].

Both cardiomyopathy and anthracycline-induced cardiomyopathy share complex molecular mechanisms, [18] including oxidative stress, mitochondrial dysfunction, calcium dysregulation, and cell death, ultimately leading to cardiac dysfunction [19]. Alternative splicing plays a crucial role in cardiomyopathy by regulating key structural and functional proteins in the heart. In dilated cardiomyopathy (DCM), splicing of Titin (*TTN*) generates different isoforms, with the shorter, stiffer N2B isoform being optimal for adult heart function, while the longer, more compliant N2BA isoform predominates in fetal hearts and during heart failure [20]. Mutations in RNA binding motif protein 20 (RBM20), a key splicing regulator, shifts the balance toward N2BA, reducing cardiac contractility and contributing to DCM [21–24]. Changes in the splicing of *OPA1*, a gene regulating mitochondrial dynamics, can shift cardiomyocytes towards a pro-apoptotic phenotype, exacerbating cell death [25]. Li, et al. showed that calcium channel CaV1.2 is alternatively spliced in diabetes-induced cardiomyopathy [26]. *CELF4* (CUGBP Elav-Like Family Member 4) is a member of the CELF family of RNA-binding proteins, which are key regulators of alternative splicing. It plays a critical role in developmental and tissue-specific splicing, especially in the heart. In the heart, *CELF4* influences the splicing of key structural and contractile proteins, like TNNT2 (cardiac troponin T), which are essential for normal cardiac function. In transgenic *MHC-CELFΔ* mice, which express a dominant-negative CELF protein in the heart, impaired CELF activity leads to early-onset alternative splicing defects, resulting in cardiac hypertrophy, dilated cardiomyopathy, fibrosis, severe heart dysfunction, and premature death [27]. The condition is more pronounced in females, indicating possible sex-specific regulation of splicing. Importantly, restoring CELF function by overexpressing CUG-BP1 (a functional CELF protein) reverses the splicing defects and heart issues, confirming that CELF activity is vital for proper splicing and cardiac function in vivo. A specific SNP, rs1786814, in the *CELF4* gene is associated with a tenfold increased risk of developing anthracycline-induced cardiomyopathy in survivors of childhood cancers, particularly in individuals with the ‘GG’ genotype [28]. This study also revealed a significant association between the presence of the ‘GG’ genotype and the coexistence of both the embryonic and adult splicing variants of *TNNT2* in myocardial tissue from patients, suggesting that the continued expression of multiple troponin T isoforms enhances cardiotoxicity in response to treatment with high-dose anthracycline. Ragab et al., corroborated these findings that the rs1786814 polymorphism in *CELF4* may influence alternative splicing of *TNNT2*, contributing to anthracycline-related cardiotoxicity in childhood cancer survivors [29]. They also showed that ‘GG’ genotype for rs1786814 was significantly associated with decreased ejection fraction and increased end-systolic diameter, indicating impaired cardiac function. Similarly, ‘GG’ genotype of rs17736312 in *ROBO2* has been reported to be significantly associated with anthracycline-induced cardiomyopathy in survivors of childhood cancers [30]. *ROBO1* and *ROBO2* encode transmembrane Robo receptors that bind Slit ligands (SLIT). The Slit-Robo signaling pathway promotes cardiac fibrosis by interfering with the transforming growth factor-β1 [TGF-β1]/Smad pathway, resulting in disordered remodeling of the extracellular matrix and potentiating heart failure. Specifically, the Slit2-Robo1 pathway is activated in fibrotic heart tissues and acts as a cardiac fibrosis-promoting component [31, 32]. Distinct isoforms for *ROBO2* [33] are reported and alternative splicing of *ROBO1* has been shown in response to hypoxia in endothelial cells [34], albeit not in context of cardiomyopathy.

However, the role of alternative splicing in anthracycline-induced cardiomyopathy among childhood cancer survivors remains unexplored. We addressed this gap by conducting a differential splicing analysis of RNA-Seq data in anthracycline-exposed childhood cancer survivors with and without cardiomyopathy.

## Methods

### Study design

Participants were enrolled to a Children’s Oncology Group (COG) study ALTE03N1 (Key Adverse Events after Childhood Cancer, NCT00082745). The multicenter trial (through the Children’s Oncology Group) was approved by the National Cancer Institute Clinical Therapy Evaluation Program on 2/26/2004. The study was initiated at City of Hope National Medical Centre as coordinating center in October 2003 and moved to University of Alabama at Birmingham in January 2015. The participants included in this study were enrolled from 11/9/2005–11/17/2017. Each participating institution obtained local ethics committee approval prior to enrolling participants. Cases consisted of childhood cancer survivors who developed cardiomyopathy after exposure to anthracyclines. COG member institutions contributed participants to the study after obtaining approval from local institutional review boards. Written informed consent/assent was obtained from patients and/or parents/legal guardians for participants < 18y. City of Hope (IRB-03066) and the University of Alabama at Birmingham Institutional Review Board (IRB-150115006) approved all experimental protocols and methods. All methods were performed in accordance with the ethical standards of City of Hope and University of Alabama at Birmingham Institutional Review Board and with the 1964 Helsinki Declaration. For each case, one anthracycline-exposed survivor with no signs or symptoms of cardiomyopathy was randomly selected as a control from the same COG cohort, matched on primary cancer diagnosis, year of diagnosis (± 5 years), and race/ethnicity. The matching allowed control for confounding factors, by ensuring that cases and controls are similar on key characteristics allowing for more precise estimates of association between exposure (anthracycline) and outcome (cardiomyopathy). The selected controls also needed a longer duration of cardiomyopathy-free follow-up compared with the time from cancer diagnosis to cardiomyopathy for the corresponding case. Participants provided peripheral blood samples in PAXgene blood RNA tubes for germline RNA.

As described in previous publications [2–4, 28, 30, 35–38], case definition was based on echocardiographic parameters: left ventricular ejection fraction (LVEF) ≤ 40% and/or fractional shortening (SF) ≤ 28%. Presence (or absence) of signs or symptoms suggestive of congestive heart failure were documented and patients were classified as “symptomatic or asymptomatic”. Lifetime anthracycline exposure was calculated by multiplying the cumulative dose (mg/m^2^) of individual anthracyclines by a factor that reflects the drug’s cardiotoxic potential and then summing the results [39]. Radiation to the chest with the heart in the field was captured as a yes/no variable. A composite binary variable for cardiovascular risk factors (CVRFs) (yes [presence of any of the following: diabetes, hypertension, dyslipidemia]; no [absence of all CVRFs]) was ascertained through self-report.

### RNA isolation, library construction, sequencing, and differential gene and transcript expression

Bioinformatic processing was performed as previously described [3]. Briefly, STAR [RRID:SCR_004463] was used to align the raw RNA-Seq fastq reads to the human reference genome from Gencode (GRCh38 p7 Release M25) [RRID:SCR_014966] [40]. For individual case–control comparisons, Cufflinks [RRID:SCR_014597)] was used on the aligned reads to assemble transcripts, estimate their abundance and test for differential expression and regulation [41, 42]. Cuffmerge [RRID:SCR_015688] was utilized to generate a transcriptome assembly and merge transcript data from all the samples. Finally, Cuffdiff [RRID:SCR_001647] was utilized to test for differences in transcript expression between cases and controls. We considered adjusted *p*-values that preserved the false discovery rate (FDR) at < 0.05 to be evidence of significant differential transcript expression.

The data discussed in this publication have been deposited in NCBI's Gene Expression Omnibus and are accessible through GEO Series accession number GSE218276 (https://www.ncbi.nlm.nih.gov/geo/query/acc.cgi?acc=GSE218276).

### Detection of differential splicing by rMATS

rMATS (v4.1.2, Update 12/17/2021; https://rnaseq-mats.sourceforge.io/rmats4.1.2/)) was used for alternative splicing analysis of the sorted, aligned sequence files [43–45]. rMATS was utilized in a fixed mode to detect five known and annotated splice events: A3'SS, A5'SS, MXE, RI and SE. Two versions of the rMATS results were generated. One version evaluated splicing variants with only those reads that spanned splice junctions (JC) and the second version included reads that spanned splice junctions and additionally those reads placed fully on the adjacent, alternatively spliced exon region (reads on target) (JCEC). rMATS quantifies splicing events as PSI (Percentage Spliced In), which is a ratio of reads specific to exon inclusion isoforms divided by the sum of reads specific to exon inclusion and exclusion isoforms. rMATS calculates the difference between PSI values (ΔPSI) between two groups under study, which serves as an effect size measure. rMATS uses a likelihood-ratio test to assess the statistical significance of ΔPSI between two groups, providing *p*-values and false discovery rate (FDR). The raw rMATS results were filtered using an FDR threshold of < 0.05 and inclusion level difference or delta percent spliced in (ΔPSI) > 0.1 or ΔPSI < −0.1 as the cutoff. For SE, A5'SS, A3'SS and RI, the results were imported based on JCEC. For MXE, the results were imported based on JC.

### Visualization of alternative splicing in integrative genome browser

Aligned reads from participants were visualized by Sashimi plots generated using IGV genome browser [RRID:SCR_011793] [46, 47]. Read densities across exons and junction reads were plotted as ‘arcs’ that were annotated with the raw number of junctions reads present in each sample.

### Gene and transcript prioritization

Prioritization was based on genes and respective transcript expression levels obtained for whole blood from GTEx portal [48]. We followed a conservative cutoff with stringent thresholds as our samples contained no replicates. Highly expressed transcripts with transcript per million (TPM) values ≥ 20 were examined further to see if alternative splicing events correlated with differential transcript expression. To evaluate the biological plausibility of the transcripts deemed differentially expressed between cases and controls, strength of evidence from literature was utilized. Tissue- and cell-specific isoform expression for each gene in human tissues was obtained from the GTEx Portal (http://gtexportal.org) and GTEx Analysis Release V8 (dbGaP Accession phs000424.v8.p2).

### Gene set enrichment analyses

We performed the Gene-Disease Association dataset (GAD) disease enrichment analyses via the Database for Annotation, Visualization, and Integrated Discovery (DAVID) tool (RRID:SCR_001881; https://david.ncifcrf.gov/home.jsp) using default settings [49, 50].

## Results

### Patient characteristics

The median age at primary cancer diagnosis for the 32 cases and 32 matched controls was 9.5 years and 10.7 years, respectively (Table 1). Cases received a higher dose of chest radiation (1335.6 cGy *vs.* 524.1 cGy; *P* = 0.04) and were more likely to have had a CVRF (37.5% *vs.* 3.1%; *P* < 0.001). The median time between cancer diagnosis and cardiomyopathy for cases was 2.4 years (IQR: 0.6–7.7); controls were followed for a significantly longer period (median, 9.4 years; *P* < 0.001).

**Table 1:** Characteristics of anthracycline-exposed childhood cancer survivors

Variables	Cases (*N* = 32)	Controls (*N* = 32)	*p*-value^a^
**Age at primary cancer diagnosis in years**			
Median (IQR)	9.5 (3.8–14.7)	10.7 (3.9–15.1)	0.71
**Sex, N (%)**			
Female	19 (59.4)	16 (50.0)	0.45
Male	13 (40.6)	16 (50.0)	
**Cumulative anthracycline exposure, N (%)**			
< 250 mg/m^2^	13 (40.6)	20 (62.5)	0.08
≥ 250 mg/m^2^	19 (59.4)	12 (37.5)	
**Chest Radiation**			
Yes (N, %)	13 (40.6)	6 (18.8)	0.05
Dose in cGy (Mean ± SD)	1335.6 ± 1953.7	524.1 ± 1185.5	**0.044**
**Race/Ethnicity (N, %)**			
Non-Hispanic white	15 (46.9)	15 (46.9)	Matched
Hispanic	9 (28.1)	9 (28.1)	
Black/African American	5 (15.6)	5 (15.6)	
Asian	3 (9.4)	2 (6.3)	
Mixed race/ethnicity	0 (0.0)	1 (3.1)	
**Primary Diagnosis, N (%)**			
Acute lymphoblastic leukemia	7 (21.9)	7 (21.9)	Matched
Acute myeloid leukemia	2 (6.3)	2 (6.3)	
Ewing sarcoma	4 (12.5)	4 (12.5)	
Hodgkin lymphoma	4 (12.5)	4 (12.5)	
Neuroblastoma	4 (12.5)	4 (12.5)	
Non-Hodgkin lymphoma	5 (15.6)	5 (15.6)	
Osteosarcoma	4 (12.5)	4 (12.5)	
Soft tissue sarcoma	2 (6.3)	2 (6.3)	
**CVRF, N (%)**			
No	18 (56.3)	31 (96.9)	**0.0006**
Yes	12 (37.5)	1 (3.1)	
Missing	2 (6.3)	0 (0.0)	
**Time from diagnosis to cardiac event for cases or time to enrollment for controls in years**			
Median (IQR)	2.4 (0.6–7.7)	9.4 (6.4–13.7)	**0.0001**

*Abbreviations*: *SD* standard deviation, *cGy* centi-gray, *IQR* interquartile range, *CVRF* Cardiovascular Risk Factors

^a^*P*-values were estimated using either chi‐square or Fisher exact test for categorical variables and the Wilcoxon/Kruskal–Wallis test for continuous variables

Values in bold indicate statistical significance at *P* < 0.05

### Summary of alternative splicing events

A total of 45 alternative splicing events were identified in 36 genes. Difference between PSI values (ΔPSI) between Cases and Controls, serves as an effect size measure which is shown as ‘Inclusion level difference’ (Table 2). SE events were the most frequent alternative splicing events, followed by RI, A5SS and A3SS events; only one MXE event was detected (Table 2). Six alternative splicing events (*BISPR*, *PPP3 CB-AS1*, *CD27-AS1*, *SNHG8* and *LINC00892*) were in long-ncRNAs and one in a pseudogene (*CASP4LP*), while the remaining 38 events were in the protein-coding genes (84.4%).

**Table 2:** Thirty-six genes exhibiting evidence of alternative splicing

Alternative Splicing Event	Gene Symbol	Biotype	GTEx Portal		rMATs		
			**Gene Expression Whole Blood (Median TPM)**	**Transcript Expression Whole Blood (Max TPM)**	**Inclusion Level Difference (All Cases vs. All Controls)**	***p*-value**	**FDR adjusted *p*-value**
**Skipped Exon**	*FASTKD3*	Protein coding	1.5	< 1	0.159	0.00E + 00	0.0000
	*BISPR*	lncRNA	4.7	1.7	0.157	2.37E-05	0.0096
	*RPGR*	Protein coding	1.7	< 1	0.128	1.53E-12	0.0000
					0.124	1.23E-12	0.0000
	*MYO15B*	Protein coding	22.6	10	0.123	3.35E-11	0.0000
	*ZNF266*	Protein coding	10.4	1	0.121	1.63E-11	0.0000
					0.115	1.36E-11	0.0000
	*EXOC3*	Protein coding	22.6	7	0.106	0.00E + 00	0.0000
	*GLT1D1*	Protein coding	84.6	17	0.105	2.24E-06	0.0013
	*PPP3 CB-AS1*	lncRNA	1	< 1	0.104	7.05E-11	0.0000
	*ZNF266*	Protein coding	10.4	1	0.103	5.74E-06	0.0029
	***PFDN5***	Protein coding	121.4	**58**	−0.109	6.23E-14	0.0000
					−0.113	7.41E-14	0.0000
	***RBM38***	Protein coding	204.7	**44**	−0.117	1.31E-09	0.0000
	*TAL1*	Protein coding	6.9	2	−0.12	3.54E-11	0.0000
	*CD27-AS1*	lncRNA	3.8	1.9	−0.121	5.55E-15	0.0000
	*ODF2L*	Protein coding	1.3	< 1	−0.124	0.00E + 00	0.0000
	*LCORL*	Protein coding	0	< 1	−0.136	0.00E + 00	0.0000
	*SLC25 A16*	Protein coding	1.2	< 1	−0.141	1.90E-06	0.0011
	***CD300A***	Protein coding	199.2	**52**	−0.142	9.94E-05	0.0321
	*SNHG8*	lncRNA	10.7	3.7	−0.156	3.88E-06	0.0021
					−0.168	4.59E-06	0.0024
					−0.171	8.56E-06	0.0042
	*NPL*	Protein coding	37.3	8	−0.184	1.03E-10	0.0000
	*CASP4LP*	pseudogene	< 1	< 1	−0.185	7.99E-05	0.0268
	*WBP1*	Protein coding	33.3	6	−0.188	3.06E-05	0.0118
	*CD27-AS1*	lncRNA	3.8	1.9	−0.206	9.21E-12	0.0000
					−0.212	4.67E-11	0.0000
**Retained Intron**	*SHMT2*	Protein coding	17.5	2	0.161	1.18E-14	0.0000
	*CAPRIN2*	Protein coding	2.2	< 1	0.126	1.59E-05	0.0008
	*ZNF517*	Protein coding	1.8	< 1	0.108	1.49E-09	0.0000
	***DDX3X***	Protein coding	49	**20**	0.107	3.65E-14	0.0000
	*CFAP410*	Protein coding	7	< 1	−0.107	2.35E-06	0.0002
	*MIB2*	Protein coding	13.2	2	−0.114	7.85E-05	0.0035
	*RPAIN*	Protein coding	3.2	< 1	−0.128	1.13E-04	0.0049
	*LINC00892*	lncRNA	< 1	< 1	−0.143	2.12E-03	0.0448
	***RPS24***	Protein coding	21.3	**37**	−0.147	9.50E-09	0.0000
	*SNRPE*	Protein coding	6.5	3	−0.179	0.00E + 00	0.0000
	*KRIT1*	Protein coding	1.8	< 1	−0.243	9.00E-08	0.0000
**MXE (JC)**	*TAZ*	Protein coding	48.1	7	−0.108	6.61E-05	0.0207
**A5**′**SS**	*TMEM134*	Protein coding	3.7	1	−0.152	1.35E-04	0.0174
	*POP5*	Protein coding	7.1	2	−0.12	4.30E-04	0.0462
**A3**′**SS**	***DDX3X***	Protein coding	49	**20**	0.1	1.08E-14	0.0000
	*TRRAP*	Protein coding	2.9	< 1	0.1	1.65E-08	0.0000
	*NCOA1*	Protein coding	17.9	3	0.121	0.00E + 00	0.0000

*Abbreviations*: *GTEx* Genotype-Tissue Expression, *rMATS* replicate Multivariate Analysis of Transcript Splicing, *MXE* Mutually Exclusive Exon, *A3'SS* Alternative 3′ Splice Site; Alternative 5′ Splice Site; *FDR* False Discovery Rate, *JC* Junction counts, *TPM* Transcript Per Million

Bold font indicates TPM ≥ 20

### Functional annotation

Table 2 shows the results from the GAD analysis to identify human diseases associated with the 36 genes. Twenty-one of the 36 genes showed a disease association. The disease classes showing relevant associations included ‘CARDIOVASCULAR’, ‘METABOLIC’ and ‘PHARMACOGENOMIC’. ‘Myocardial Infarction’, ‘Cholesterol’, ‘Diabetes’, ‘Coronary Artery Disease’ and ‘Cardiomyopathy’ were the most relevant diseases associated with the alternatively spliced genes (Table 3).

**Table 3:** Disease annotation of 21 (of 36) genes using the Gene-Disease Associations dataset (GAD) from Database for Annotation, Visualization, and Integrated Discovery (DAVID)

AS Event	Gene Name	GAD_Disease_Class	GAD_Disease
**Skipped Exon**	*CD300A*	**Cardiovascular**	**Myocardial Infarction**
	*FASTKD3*	**Pharmacogenomic**, Renal	Chronic renal failure, **Type 2 Diabetes**
	*NPL*	**Metabolic**	**Cholesterol, HDL**
	*RBM38*	**Cardiovascular**, Hematological,** Metabolic**	**Cholesterol**, Erythrocyte Indices, Forced Expiratory Volume, Forced Vital Capacity, **Myocardial Infarction**
	*TAL1*	Cancer, **Pharmacogenomic**	**Type 2 Diabetes**, myeloid leukemia,
	*GLT1D1*	**Cardiovascular**, Chemdependency, Immune	Monocytes, **Myocardial Infarction**, Tobacco Use Disorder
	*LCORL*	Chemdependency, Developmental, Normal Variation	Height, Tobacco Use Disorder, skeletal frame size
	*ODF2L*	**Cardiovascular**	Forced Expiratory Volume
	*RPGR*	Vision	Retinal Diseases, Retinitis Pigmentosa, retinal dystrophy
	*SLC25 A16*	Chemdependency, Infection, Neurological	Acquired Immunodeficiency Syndrome, Alzheimer's disease, Tobacco Use Disorder
	*ZNF266*	Immune	Crohn's disease
**Retained Intron**	*DDX3X*	Infection	HIV Infections
	*KRIT1*	**Cardiovascular**, Chemdependency	Tobacco Use Disorder, cerebrovascular disease, intracranial cavernous malformations
	*RPAIN*	Reproduction	Patent ductus arteriosus
	*CAPRIN2*	**Cardiovascular**	**Coronary Artery Disease**, **Electrocardiography**
	*RPS24*	**Cardiovascular**, Hematological, Metabolic, Psych	Diamond-Blackfan anemia, **Cholesterol, HDL**, **Conduct Disorder**, **Electrocardiography**, Erythrocyte Count, Metabolism, **Myocardial Infarction**, Schizophrenia
	*SHMT2*	Cancer, Developmental, Infection, **Metabolic**, **Pharmacogenomic**, Psych	1-carbon metabolism, Acquired Immunodeficiency Syndrome, Cleft Palate, Lymphoma, Non-Hodgkin, **Type 2 Diabetes**, neural tube defects, several psychiatric disorders
**Mutually Exclusive Exon**	*TAFAZZIN*	**Cardiovascular, Metabolic**	**Cardiomyopathy**, **Dilated cardiomyopathy**, Thyroid Dysgenesis
**A5**′**SS**	*TMEM134*	Vision	Macular Degeneration
**A3**′**SS**	*TRRAP*	**Cardiovascular**, Chemdependency, **Metabolic**	**Insulin Resistance**, Receptors, Tumor Necrosis Factor, Tobacco Use Disorder
	*NCOA1*	Cancer, **Cardiovascular**, Chemdependency, Immune, **Metabolic, Pharmacogenomic**	Bone Mineral Density, Heart Rate, Tobacco Use Disorder, **Type 2 Diabetes, arterial blood pressure**, breast cancer, bronchodilator response, epithelial ovarian cancer, plasma **HDL cholesterol** (HDL-C) levels, prostate cancer

Bold font indicates cardiac‐related categories

### Prioritization strategy for alternative splicing events

We excluded genes with a TPM value < 20 of the most abundant transcript/isoform in whole blood. We checked the remaining genes (*PFDN5*, *RBM38*, *CD300A*, *DDX3X* and *RPS24*) manually using IGV to identify genes with a change in expressed alternative splicing patterns between the cases and controls. To this end, we tested if there was a visible difference in used splice sites based on junction information, or a change in exon and intron expression. We checked whether the observed alternative splicing event was visually dominant in cases *vs.* controls and if differences in inclusion levels or inclusion junction counts (IJC) and skipped junction counts (SJC) for each case/control pair correlated with differential transcript expression. Alternative splicing events in two genes: *RPS24* and *PFND5* met the above criteria.

**Fig. 1:**
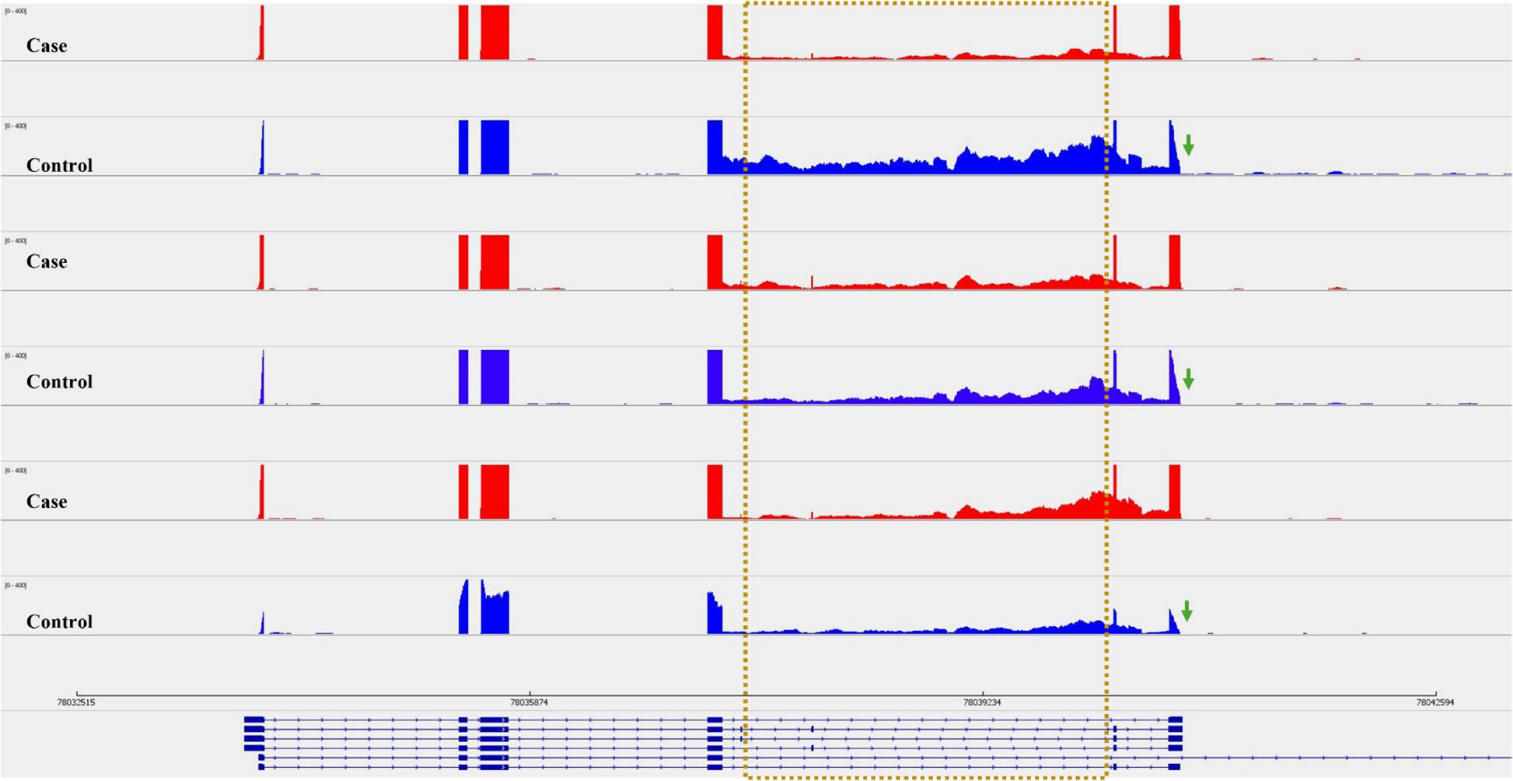
Sashimi plots in IGV genome browser depicting retained intron in *RPS24* for representative samples. Cases are shown in red and matched controls are shown in blue. Genomic coordinates are plotted on x-axis and read density (whose value is configurable via IGV) on y-axis, and mRNA isoforms quantified are shown on bottom (exons in blue, introns as lines with arrow heads). Data for the plot were taken from three sets of paired samples. The plot highlights the differential splicing of the intron, which is present in the controls, but mostly absent in the case samples. Exon coverage max set to the same level for all samples. Junction coverage minimum >10. It is interesting to note that there is an abrupt decrease in the density of last exon in controls (marked by arrows)

**Fig. 2:**
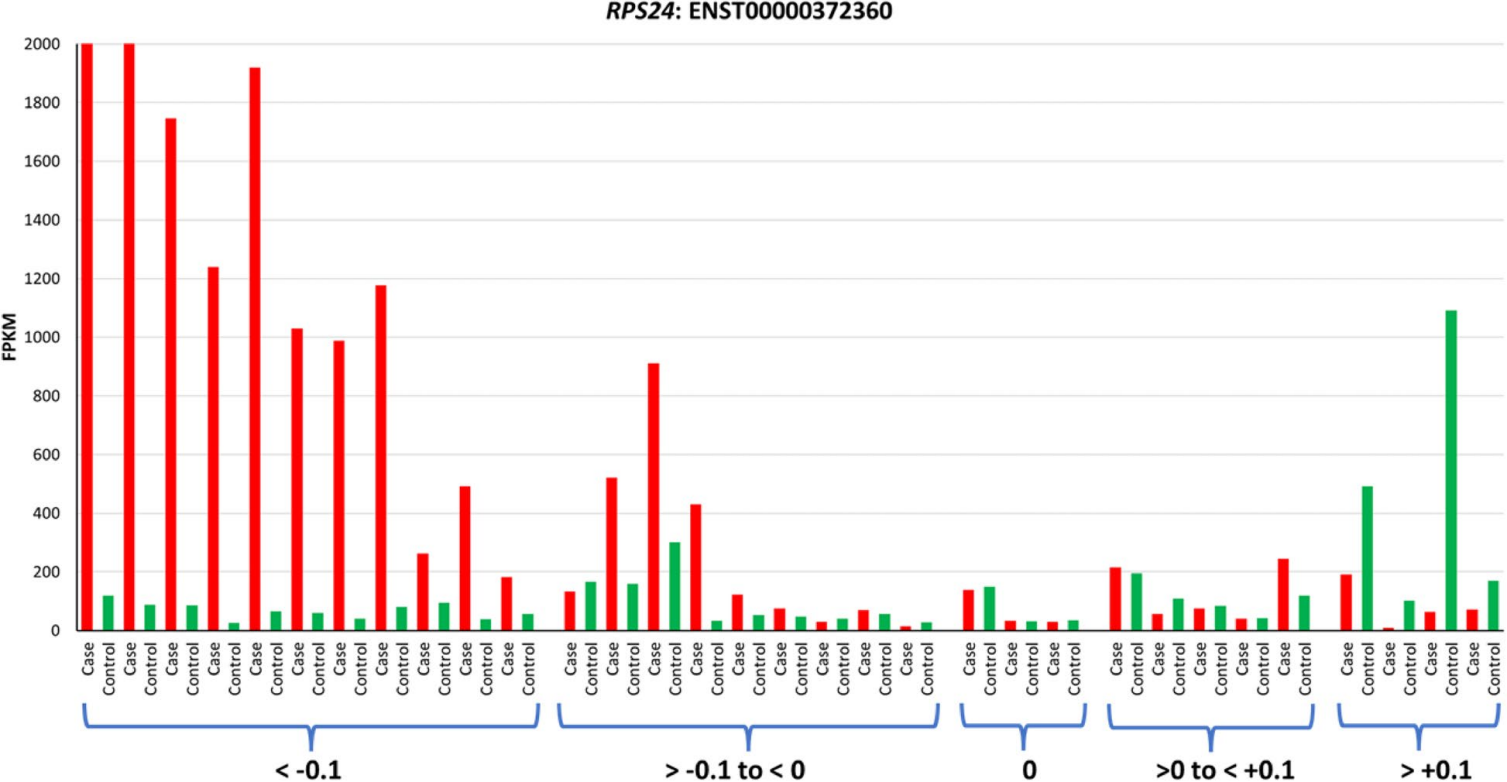
Intron retention level is inversely correlated with the differential expression of *RPS24* transcript ENST00000372360. The expression level of the transcript measured as FPKM (Fragments per Kilobase of transcript per Million mapped reads) is plotted on the Y-axis. The matched case–control sets are plotted side by side on the X-axis. Cases are indicated in red, and control subjects are indicated in green. The samples are ordered by pairwise inclusion level difference from rMATS to show correlation with transcript expression. Inclusion level difference of < −0.1 indicates that intron inclusion level in cases is lower compared to controls and inclusion level difference of > + 0.1 indicates higher level of inclusion in cases compared to controls. Transcript ENST00000613865 showed a similar pattern, albeit at lower FPKM values

Sashimi plots depicting retained intron in *RPS24* (S1 Fig) for representative samples are shown in Fig. 1. We found that the intron retention level was inversely correlated with the differential expression of *RPS24* transcript ENST00000372360. Overall controls showed a higher level of intron retention compared to cases (Fig. 2). Most mRNA derived from retained intron event is degraded via nonsense-mediated decay (NMD) resulting in the observed decreased expression of the transcript in controls compared to cases. However, we observed that the alternative splicing event mainly caused dysregulation of *RPS24* at transcript level rather than gene expression level (S1 Table and S3 Fig).

**Fig. 3:**
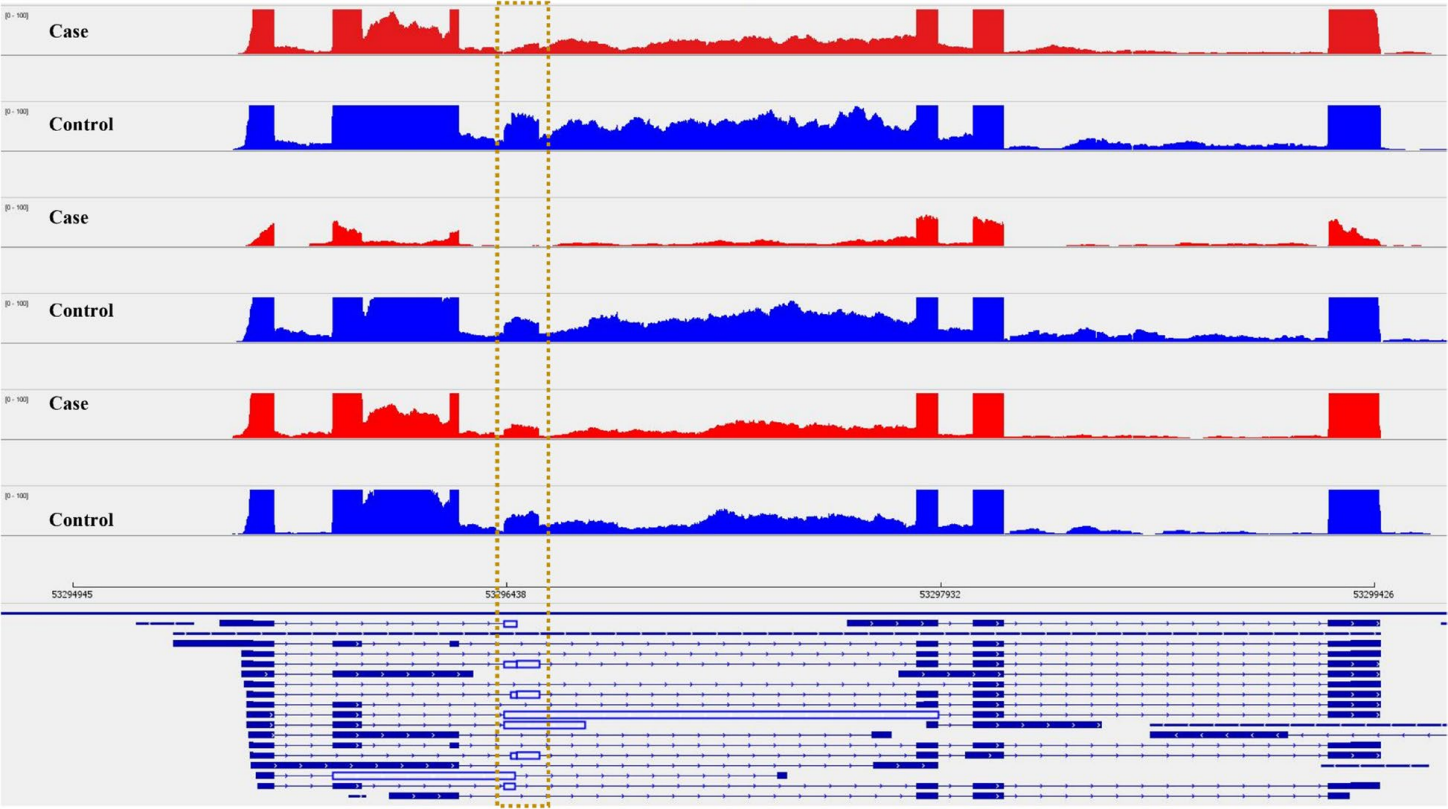
Sashimi plots in IGV genome browser depicting exon skipping in *PFDN5*. The plot highlights the differential splicing of the exon, which is largely present in the controls, but mostly absent in the case samples. Exon coverage max set to the same level for all samples

**Fig. 4:**
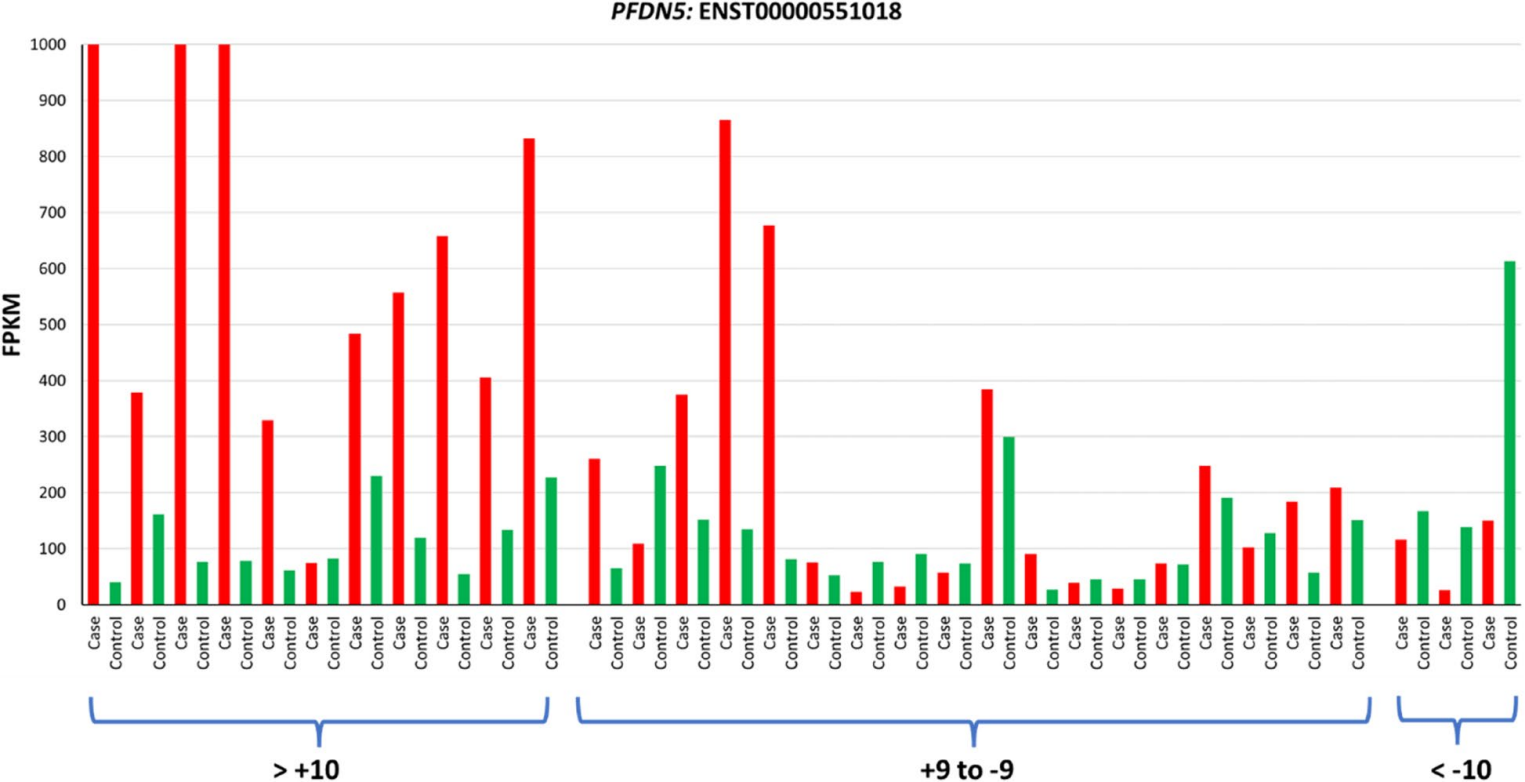
Correlation between difference in IJC in paired cases and controls and transcript expression for *PFDN5* transcript ENST00000551018. The expression level of the transcript measured as FPKM is plotted on the Y-axis. The matched case–control sets are plotted side by side on the X-axis. Cases are indicated in red, and control subjects are indicated in green. The samples are ordered by pairwise inclusion junction count (IJC) difference from rMATS to show correlation with transcript expression

Exon skipping was observed in *PFDN5* (Fig. 3 and S2 Fig). ENST00000551018, the transcript with skipped exon showed higher expression in cases compared to controls (Fig. 4). The alternative splicing event caused dysregulation of *PFDN5* both at transcript level and at gene expression level (Fig. 4 and S1 Table).

## Discussion

Aberrant alternative splicing has been widely reported in a variety of disease states in the general population, including heart failure [13–15]. We performed an exploration of alternative splicing in whole blood samples from anthracycline-exposed childhood cancer survivors with and without cardiomyopathy, which enabled a quantitative assessment of alternative splicing events and a qualitative identification of genes affected by alternative splicing. We identified intron retention in *RPS24* and exon skipping in *PFDN5* with expression-level differences for transcripts harboring retained introns and skipped exons. *RPS24* transcript ENST00000372360 and *PFDN5* transcript ENST00000551018 had higher expression in patients with cardiomyopathy with direct correlation of intron retention and exon skipping to lower transcript level expression in controls. While *RPS24* and *PFDN5* are not traditionally recognized as major cardiac genes, their roles in protein synthesis, folding, and cellular stress responses suggest they could influence cardiac physiology and pathology.

Intron retention is an alternative splicing method whereby introns, rather than being spliced out, are retained in mature mRNAs. Intron retention interrupts the main open reading frame and may lead to inclusion of premature termination codons, whereby intron-retaining isoforms are often rapidly degraded by the NMD pathway, resulting in downregulation or no gene expression [51]. Exon skipping or ES is the most common alternative splicing event whereby a particular exon is omitted from an alternatively spliced mRNA [52].

Ribosomes are a complex of ribosomal RNA and ribosomal proteins that function as a machinery for mRNA translation and protein synthesis. Elevated protein synthesis rates are characteristic of proliferating cells that need new cellular constituents [53]. The eukaryotic ribosome enzyme system is composed of four ribosomal RNAs (rRNAs) and 80 ribosomal proteins (RPs). The 40S ribosomal protein S24 (*RPS24*) is one of the ribosomal proteins. Studies have shown that RPs have additional ribosomal functions including roles in DNA repair, replication, proliferation, apoptosis and chemoresistance [54]. Ribosomal dysfunction has been implicated in a variety of developmental disorders including congenital heart disease [55]. Several RPs have been implicated in the development and progression of cardiovascular disease in the general population [56, 57]. The Minute syndrome in *Drosophila*, is associated with RP haploinsufficiency, and is characterized by developmental delay, impaired growth, poor fertility, and cardiac dysfunction [58]. RNAi-mediated knockdown of RpS24 in *Drosophila* cardiac tissue reveals that the hearts of these larvae cease to contract within 48 h with accompanying cardiac atrophy and breakage of cardiac collagen [58]. These phenotypes suggest that RpS24 is essential for cardiac integrity. Interestingly, mutations in *RPS24* cause Diamond Blackfan anemia; with a high prevalence of congenital heart disease (~ 30%) [59, 60]. In a previous study, characterization of mCpG in heart failure showed that *RPS24* is associated with heart failure. [61] Kerry et al., have recently shown that alternative splicing of *RPS24* results in long vs. short variants, where the long variant produces a more stable protein isoform that aids in hypoxic cell survival. [62] A splice altering variant in *RPS24* (chr10:79800375G > A) [63] has been identified in patients with atrial septal defect, another congenital heart defect.

The Prefoldin Subunit 5 (*PFDN5*) encodes one of the six subunits of prefoldin, a molecular chaperone complex that binds and stabilizes newly synthesized polypeptides and regulates the folding of nascent actin and tubulin monomers, essential for cardiomyocyte integrity [64]. Splicing alterations in *PFDN5* may impair the proper folding of actin and tubulin, leading to cytoskeletal abnormalities that contribute to heart failure and arrhythmias. Furthermore, since PFDNs contribute to cellular adaptive response to stress, its mis-splicing could disrupt cellular stress responses, making cardiomyocytes more susceptible to ischemic damage [65, 66]. Zhang et al. [67], showed that *PFDN5* was upregulated in patients with chronic heart failure and is a promising biomarker for the prediction of heart failure. Similar to our study, Li et al. [68], showed that both *RPS24* and *PFDN5* were upregulated in individuals from the general population with heart failure. Chen et. al., identified *RPS24* and *PFDN5* as key hub genes that are dysregulated in hypertrophic cardiomyopathy samples compared to healthy controls [69]. Additionally, whole exome sequencing data showed that *RPS24* mutations were associated with heart failure.

Hypoxia is a key regulator of cardiac hypertrophy and hypoxia also induces hypoxia-inducible factor 1-alpha (*HIF1A*) that in turn induces alternative splicing [34]. Alternative splicing events in *RPS24* transcripts have been reported to be altered by hypoxia and favors hypoxic cell survival [70]. In mammals, the PFDN complex including *PFDN5* binds to nascent actin and tubulin cytoskeletal proteins to deliver them to the chaperonin CCT to promote their folding [65]. In the heart, both actin and tubulin are crucial components of the cytoskeleton, playing vital roles in cardiac function and structure, with actin forming the sarcomeric units for contraction and tubulin forming microtubules that support cell shape and transport. In cardiomyopathy, the actin and tubulin cytoskeleton undergo remodeling, including changes in the density, stability, and post-translational modifications of microtubules [71]. Splicing alterations resulting in differential expression of splicing isoforms in *RPS24* and *PFDN5* could compromise protein homeostasis, cardiac structure, and stress response, ultimately contributing to cardiomyopathy (Central Illustration).

Many studies have examined alternative splicing in cardiovascular diseases [27, 28, 72, 73] and various genome editing and small molecules have been demonstrated to be able to correct alternative splicing patterns [74–76]. Manipulating dysregulated RNAs to adjust expression could improve the course of anthracycline-induced cardiomyopathy [76]. Identifying specific pathological alternative splicing targets is key to offering new insights into their therapeutic potential.

## Limitations

Ideally, alternative splicing should be measured in the affected tissue (i.e., cardiac tissue). However, obtaining heart biopsies from cancer survivors is logistically challenging and not without risk. Recent evidence has shown that peripheral blood reflects the transcriptomic signature of other tissues including heart [77–81]. Prevalent case–control studies by the very nature of their design exclude fatal endpoints from the case set. Presence of survival bias risks under-ascertainment of genes associated with high lethality, with consequent underestimation of disease risk effect size for their alternative splicing events associated with both increased disease risk and disease-associated lethality. Further research should focus on replicating current findings in a larger sample size.

## Conclusions

Our findings provide an insight into the altered splicing landscape of anthracycline-induced cardiomyopathy. We show that alternative splicing is a prominent feature in the blood obtained from patients with anthracycline-induced cardiomyopathy. We identified alternative transcripts of *RPS24* and *PFDN5* genes among childhood cancer survivors with anthracycline-induced cardiomyopathy. These genes have been previously implicated in the pathogenesis of cardiovascular diseases in the general population. Anthracycline-induced cardiomyopathy is a complex disease, where the relationship between gene expression and phenotypes is subject to various genetic and epigenetic influences prior to its clinical manifestation; examining alternative splicing events adds a piece to the whole puzzle. We therefore propose that examining alternative splicing should be included as part of the gene expression analysis as it provides additional insight into the transcriptomic landscape and could potentially allow a more accurate prediction of the functional consequences of detected changes in gene expression.

## Supplementary Information

Supplementary Material 1

## Data Availability

The data discussed in this publication have been deposited in NCBI's Gene Expression Omnibus and are accessible through GEO Series accession number GSE218276 https://www.ncbi.nlm.nih.gov/geo/query/acc.cgi?acc = GSE218276.

## Abbreviations

COG: Children’s Oncology Group
mRNA: Messenger RiboNucleic Acid
RNA-Seq: RNA Sequencing
AS: Alternative Splicing
DGE: Differential Gene Expression
A5SS: Alternative 5′ Splice Site
SE: Skipped Exon
MXE: Mutually Exclusive Exon
RI: Retained Intron
A3SS: Alternative 3′ Splice Site
rMATS: Replicate Multivariate Analysis of Transcript Splicing
PSI: Percent Spliced In
IGV: Integrative Genomics Viewer
TPM: Transcript Per Million
GTEx: Genotype-Tissue Expression
NMD: Nonsense-mediated mRNA decay
rRNA: Ribosomal RNA
FPKM: Fragments per Kilobase of transcript per Million mapped reads
