# Supplementary material for: Alternative mRNA splicing in anthracycline-induced cardiomyopathy – a COG-ALTE03N1 report
Source: Cardiooncology. 2025 May 17;11:47. doi: 10.1186/s40959-025-00345-2 (PMC12084991; doi:10.1186/s40959-025-00345-2)

# **Supporting information**

**S1 Table: Gene and transcript level expression of *RPS24* and *PFDN5* in cases and controls**

| **Normalized RNA counts** | **Cases (N=32)** | **Controls (N=32)** | **p-value*** |
| --- | --- | --- | --- |
| ***RPS24*** | | | |
| Median (IQR) | 1044.5 (762.3-2200.5) | 1046.6 (770.3-1602.1) | 0.07 |
| ***PFDN5*** |  |  |  |
| Median (IQR) | 2312.1 (1205.4-4482.1) | 1350.0 (1073.8-1906.5) | **0.010** |
| **FPKM** | **Cases (N=32)** | **Controls (N=32)** | **p-value*** |
| **ENST00000372360 *(RPS24)*** | | | |
| Median (IQR) | 186.9 (65.1-968.8) | 81.9.9 (41.1-141.6) | **0.016** |
| **ENST00000551018 *(PFDN5)*** | | | |
| Median (IQR) | 228.3 (74.4-538.6) | 86.1 (61.9-159.1) | **0.019** |

Abbreviations: FPKM, Fragments per Kilobase of transcript per Million mapped reads;

IQR, interquartile range. *P-values were estimated using the Wilcoxon/Kruskal-Wallis test for continuous variables.


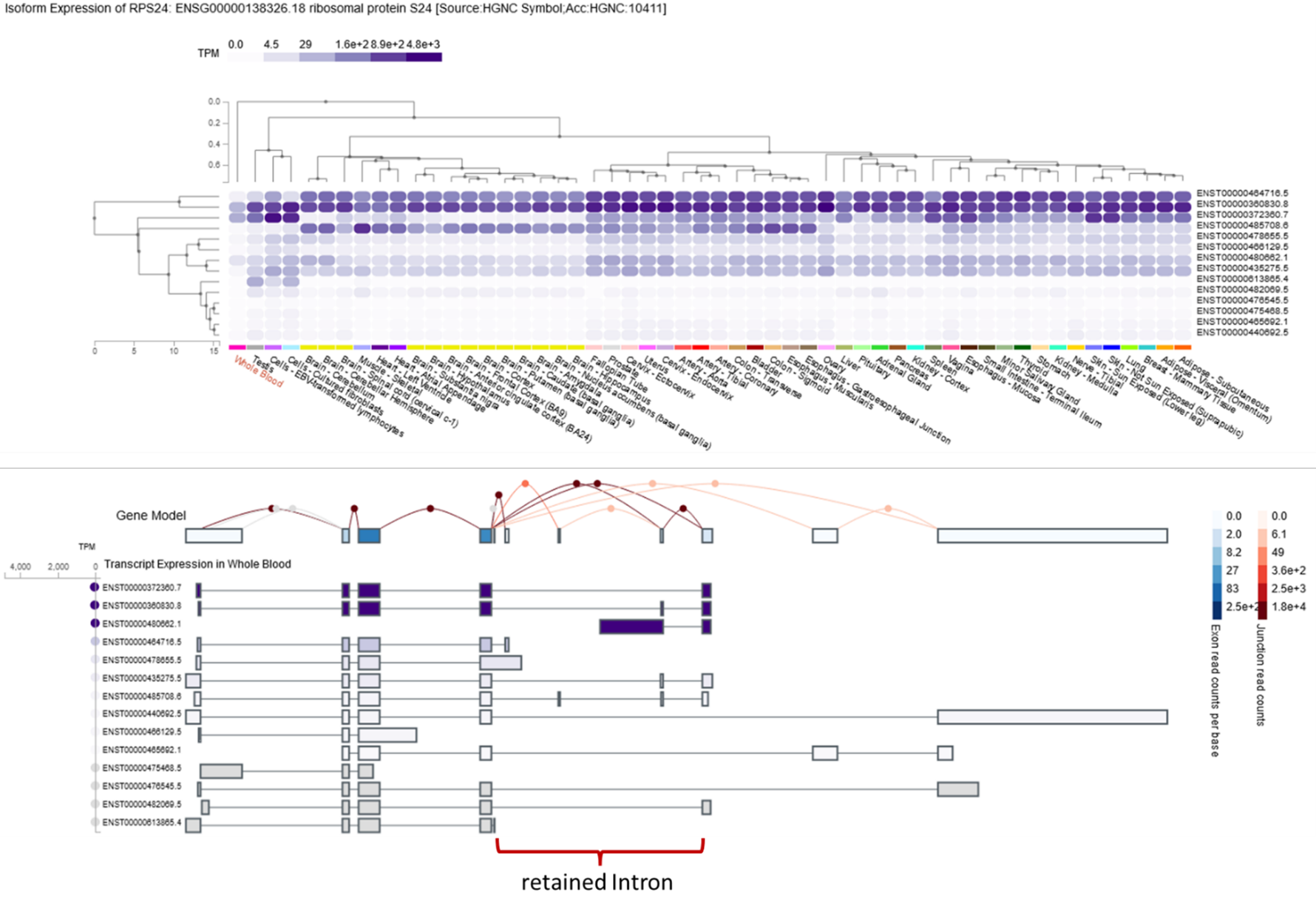
**S1 Fig**. **Tissue- and cell-specific expression for *RPS24* (ENSG00000138326.18) isoform expression in human tissues.** The median expression (TPM) of isoforms in each tissue is summarized using a heat map. The lower panel shows the exon and junction expression in whole blood. The retained intron (Start: 78037305 and End: 78040614) is shown. Data Source: GTEx Analysis Release V8 (dbGaP Accession phs000424.v8.p2).


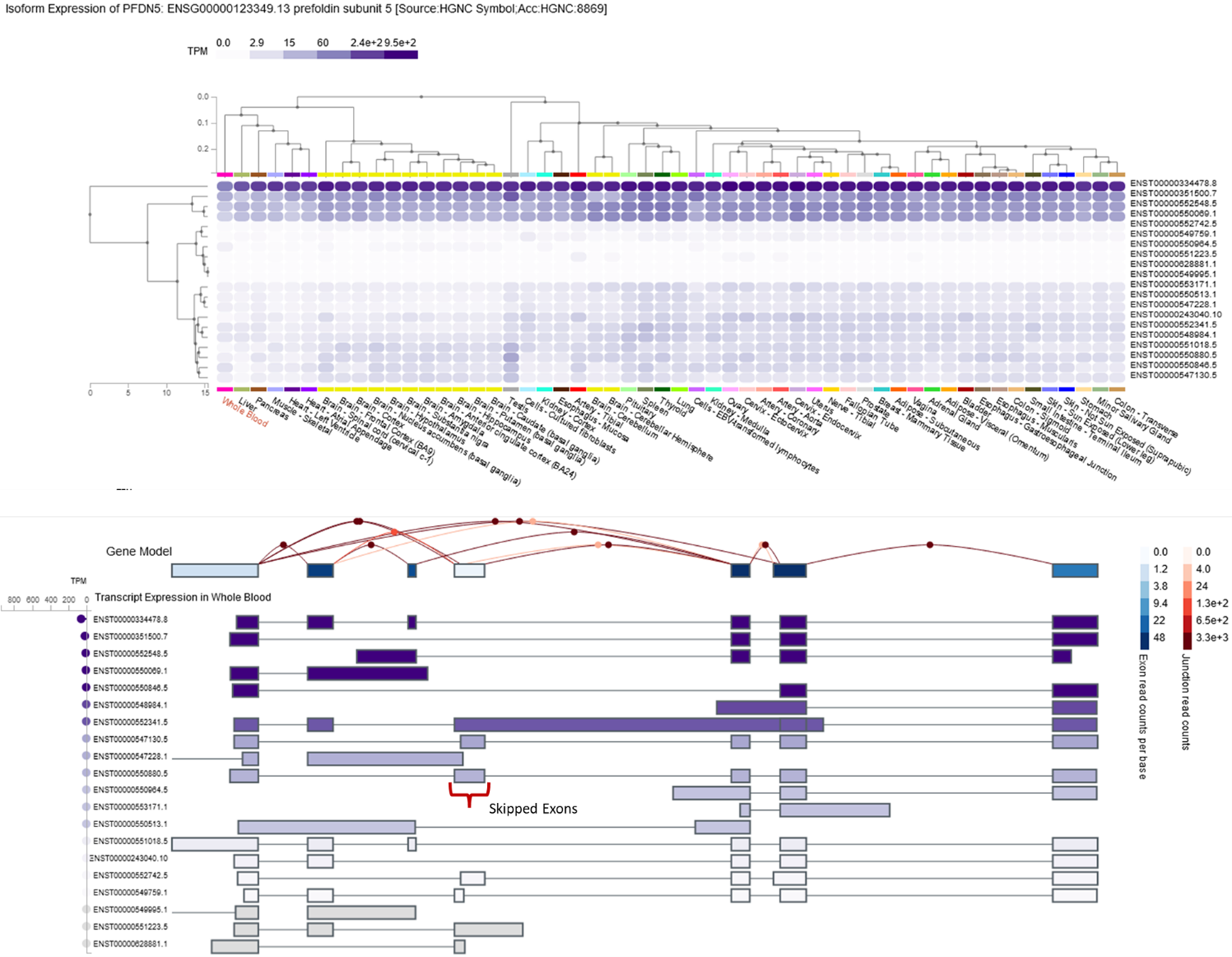
**S2 Fig**. **Isoform expression for *PFDN5* (ENSG00000123349.13) in human tissues.** The two skipped exons (Start: 53296454 and 53296430 End: 53296551) are shown. The lower panel shows the exon and junction expression in whole blood.

**S3 Fig. Intron Retention level is not correlated with the global gene expression of *RPS24.*** The normalized aggregate read RNA counts from DESeq2 for ***RPS24*** gene are shown. The matched case-control sets are plotted side by side. Cases are indicated in red, and control subjects are indicated in green.


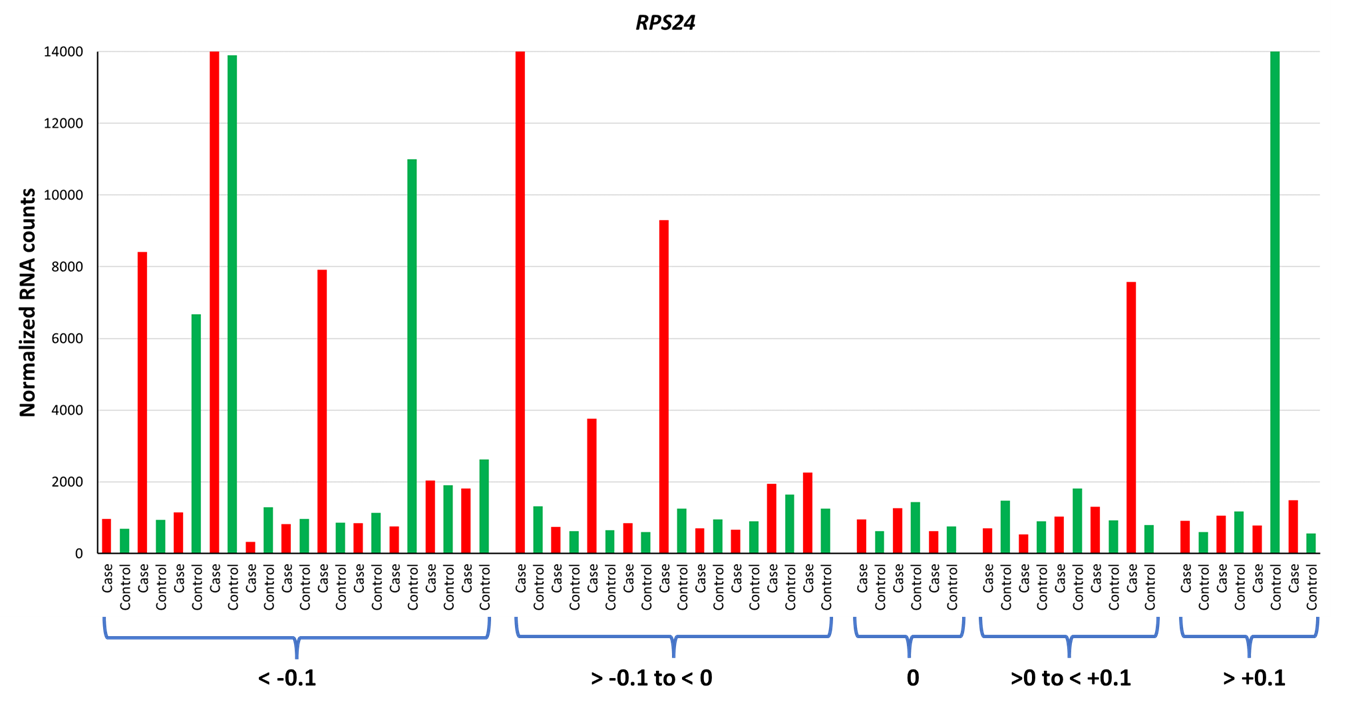


**S4 Fig. Exon skipping is correlated with the global gene expression of *PFDN5.*** The normalized aggregate read RNA counts from DESeq2 for ***PFDN5*** gene are shown. The matched case-control sets are plotted side by side. Cases are indicated in red, and control subjects are indicated in green.


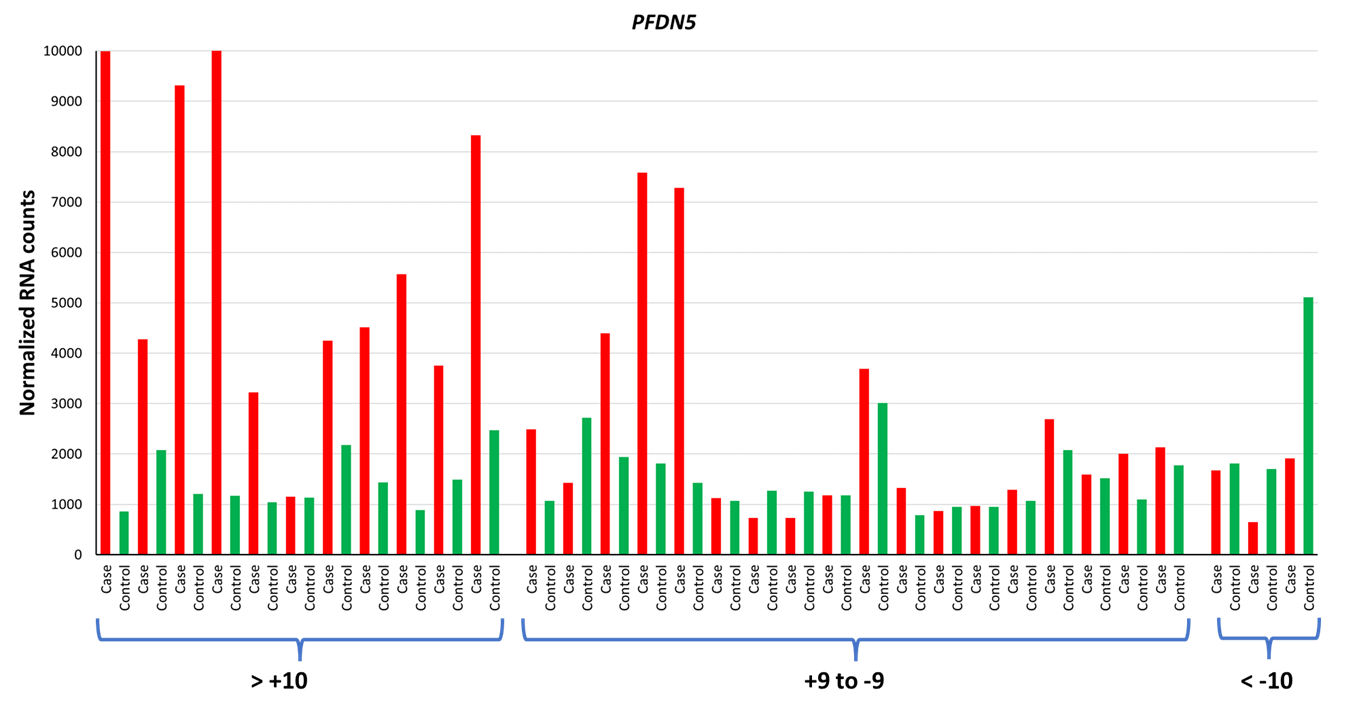

Supplement: Supplementary file 1 — Supplementary Material 1 [file 40959_2025_345_MOESM1_ESM.docx]
